# Intergenerational crosstalk of brain-gut axis in parental Nd_2_O_3_ exposure-induced offspring neurotoxicity and cognitive dysfunction: a mechanistic study

**DOI:** 10.3389/fpubh.2024.1470502

**Published:** 2024-11-12

**Authors:** Yujing Jia, Jing Cao, Yan Guo, Lihong Wu, Xiaoyan Du, Bofu Tang, Bingtao Xia, Yang Deng

**Affiliations:** ^1^Baotou Medical College, Inner Mongolia University of Science and Technology, Baotou, China; ^2^Inner Mongolia Institute of Digestive Diseases, The Second Affiliated Hospital of Baotou Medical College, Inner Mongolia University of Science and Technology, Baotou, China; ^3^Baotou Customs District P.R.China, Baotou, China; ^4^The Third Hospital of Inner Mongolia Baogang Group, Baotou, China

**Keywords:** Nd_2_O_3_, brain-gut axis, neurotoxicity, cognitive dysfunction, prenatal exposure

## Abstract

**Introduction:**

Rare earth elements (REEs) are widely used in plenty of fields. REEs have significant neurotoxicity and it may adversely affect the development of cognitive. For example, neodymium will causing neurological damage through penetrate the blood–brain barrier (BBB). However, whether it disrupts the balance of brain-gut axis (BGA) crosstalk and affects the intestinal microecology disorder of host is still unclear. This study investigated the neural damage on children caused by maternal exposure to Neodymium oxide (Nd_2_O_3_) during pregnancy, and its involved mechanism of BGA injury.

**Methods:**

We used rat model to investigated the mechanisms of the offspring’s neural damage that Nd_2_O_3_ exposure in pregnancy. To verify the neural damage of offspring rats, we examed BBB-related factors, such like glutamate and ROS levels in brain tissue, behavioral tests, hippocampal and cortical damage, as well as changes in gut microbiota, intestinal mucosal barrier, and SCFAs in the intestine. Also, we observed some specific indicators of intestinal immune barrier function and gut nerve-related indicators.

**Results:**

Maternal Nd_2_O_3_ exposure reduced the content of offspring tight junction proteins, increased BBB permeability, leading to Nd accumulation and brain tissue inflammation, affecting offspring’s neural development and weakening their spatial learning ability. Nd_2_O_3_ also disrupted BBB integrity by regulating SCFAs and BGA. Probiotic intervention in the offspring rats exposed to 2% Nd_2_O_3_ showed significant recovery of inflammation in both brain and colon tissues, and reduced BBB permeability.

**Conclusion:**

Maternal exposure to Nd_2_O_3_ affects the offspring’s BGA, targeting brain and colon tissues, increasing BBB permeability, affecting neural development, causing damage to the intestinal mucosa, and impacting children’s gut development. Probiotics can alleviate these effects. These findings provide valuable insights into understanding the neurodevelopmental and intestinal developmental toxicity of Nd_2_O_3_ and its prevention and treatment. It also calls for a comprehensive assessment of the health risks of susceptible populations to Nd_2_O_3_, such as pregnant women. It may providing theoretical basis for preventing and controlling neodymium-induced harm in children by examing the repair mechanism of the damage through probiotic intervention.

## 1 Introduction

Rare earth elements (REEs) are widely used in many fields such like agriculture, forestry, animal husbandry, fisheries, and pharmaceutical and chemical industries and so on. The environmental residues and accumulation may the core reasons that caused REEs raising significant public health concerns, especially in children (1). Currently, REEs have been detected in hair, nails, and bodily fluids of human, leading to nephrogenic systemic fibrosis and severe damage to the renal system, as well as neurological damage, fibrotic tissue injury, oxidative stress, pneumoconiosis, cytotoxicity, anti-testicular effects, and male infertility (2). Among the 17 REEs, La, Gd, Ce, and Eu have been extensively studied, while research on Nd is few (2, 3). Neodymium-iron-boron permanent magnet technology is widely used in various fields, making neodymium account for nearly 50% of the major rare earth market share in the world. However, deeply systematic research on the safety of large-scale mining and application of neodymium, particularly concerning the well-being of children living in high-neodymium exposure environments, has not been investigated. Additional, widespread mining and use of rare earths in address developmental impairments in children is urgently necessary.

Epidemiological studies conducted in several major rare earth mining areas in China have shown that the average IQ, cognitive abilities, and learning and memory capabilities of children in these areas are significantly lower than those which in control areas (4). Therefore, REEs have significant neurotoxicity and can have adverse effects on cognitive development (1–4).

Additional, nanometer neodymium oxide (Nano-Nd_2_O_3_) administered by nasal instillation has a mild stimulating effect on learning and memory in mice by affecting the activity of calcium ion channels (5). Furthermore, studies have found that Nano-Nd_2_O_3_ can lead to a decrease in the ability of brain tissue to remove lipid peroxides and an increase in oxidative stress levels in mice, demonstrating its toxic effects on the central nervous system (6). Nano-Nd_2_O_3_ disrupts the oxidation-antioxidant balance in the hippocampus and alters the levels of amino acid neurotransmitters, leading to neuronal damage and impairing learning and memory function in developing rats (7).

The first barrier that rare earth elements must overcome to damage the nervous system is the blood–brain barrier (BBB). The BBB is a multicellular complex composed of brain microvascular endothelial cells (BMECs), tight junctions (TJs) between cells, the basement membrane, and embedded pericytes and astrocyte foot processes. It can prevent certain toxic substances from entering the brain, protecting brain tissue from invasion by toxic substances and pathogens in the blood, providing nutrients to brain tissue, and maintaining the internal environment of the central nervous system (CNS) (8). The immature development of the BBB in early postnatal life provides an opportunity for metals and other toxic substances to enter brain tissue. Additionally, these substances may disrupt TJs, causing the BBB to leak and allowing more toxic substances to enter and accumulate in brain tissue, affecting the proliferation and differentiation of nerve cells and synaptic structure and function, leading to neural developmental damage. However, there have not researches about whether Nd can break through the BBB as we know.

The human gut is the “secret garden” of 10 trillion diverse symbionts, collectively known as the “microbiota.” Microbiota are 10 times more abundant than our somatic and germ line cells of the body. The collective genes of microbiota are known as the “microbiome” which is 150 times larger than the human genome (9, 10). Certain crucial traits are indispensable for microbes aiming to colonize new environments. These include a comprehensive suite of enzymes that enables them to harness accessible nutrients effectively, a precise configuration of cell-surface molecules that facilitates adherence to suitable niches within their prospective habitats. Additionally, the capacity to circumvent predation by bacteriophages and adaptiveness to counteract the vigilant immune responses of the host organism are paramount. It’s also essential for these microorganisms to possess resilience against both the physical and chemical rigors present in the gastrointestinal tract, allowing them to multiply swiftly while resisting expulsion through peristalsis (11).

Significant progress has been made in elucidating the bidirectional interactions between the nervous system, the gut, and the gut microbiota based on studies in experimental animals. Many studies have shown that the gut microbiota can regulate the structure and function of the enteric nervous system, forming a bidirectional connection with the gut-brain axis (GBA), thereby constituting the Brain-Gut-Microbiome (BGM) axis (12). The gut microbiota and its metabolites can influence the brain through neural, immune, and endocrine pathways, and the brain, in turn, regulates the composition of the gut microbiota through this pathway to maintain gut microbial balance. There have been no reports on the effects of rare earth neodymium exposure on the BGM axis in children living in rare earth mining areas. Studies have indicated that signal transduction within the BGM axis is regulated by two dynamic barriers, namely the BBB and the intestinal barrier (13).

Neodymium causes damage to the nervous system, but whether it leads to changes in the balance of BGA crosstalk, affecting the imbalance of the host’s gut microbiota and leading to changes in the BGA, has not been reported as we know. Therefore, starting from this specific group of children, it is crucial to study the effects and mechanisms of maternal exposure to Nd_2_O_3_ during pregnancy on BGA damage, and how to prevent and control the damage caused by neodymium to children, which is of great significance both at the individual and national levels. This study aims to explore the impact of neodymium on the development of the BGA in children, and thus provide a theoretical basis for the prevention and control of neodymium-induced damage during childhood.

## 2 Materials and methods

### 2.1 Animals and treatment

Sixty healthy adult female rats and sixty male rats weighing 260 ± 10 g were provided by Sbeifu (Beijing) Biotechnology Co., Ltd. and kept in the animal room for at least 1 week to adapt to the environment. The environmental temperature in the animal room was maintained at 17–23°C, with a relative humidity of 45–55%. The animal feed was provided by the Animal Experimental Center. Rats were mated in a 1:1 ratio of male to female, and the morning after mating, the presence of a vaginal plug marked as pregnancy day 0. Referring to previous research by Gao et al. (14, 15), the administration of Nd_2_O_3_ occurred during the gestation and lactation periods (22 days +21 days), with dosing frequencies of 0, 50, 100, and 200 mg/(kg·d). Pregnant rats were randomly divided into control group (distilled water of equal volume), 0.5% Nd_2_O_3_ group, 1% Nd_2_O_3_ group, 2% Nd_2_O_3_ group, and 2% Nd_2_O_3_ + BTVT (Bifidobacterium tetrad viable tablet, with the main component of Lactobacillus, Hangzhou Yuanda Biopharmaceutical Co., Ltd., 150 mg/kg) group for Nd_2_O_3_ gavage exposure during pregnancy and lactation, establishing an animal model of offspring exposure (gestation: pregnancy day 22, weaning: pregnancy day 43). All pregnant rats were fed *ad libitum* with food and water until the offspring were feed till adulthood (postnatal day 83) to conclude the animal experiment. Behavioral interventions using the BTVT model commenced on postnatal day 1 (PND 1). Given that the BBB is not yet fully developed in infants under 6 years of age and in rats within 21 days postnatally (specifically during the weaning period at PND 43), the study was conducted on pups at PND 43.

### 2.2 Nd_2_O_3_ exposure-induced neurological damage

#### 2.2.1 Detection of BBB-related factors

Ten offspring rats from each of the following groups—pregnant rats exposed to 0.5% Nd_2_O_3_, 1% Nd_2_O_3_, 2% Nd_2_O_3_, 2% Nd_2_O_3_ + BTVT, and the control group (distilled water of equal volume) - were randomly selected for BBB-related tests on PND43 (postnatal day 43). ICP-MS was used to detect the rare earth neodymium content in the rat brains, the Evans blue method was employed to observe changes in blood–brain barrier (BBB) permeability in offspring rats, and transmission electron microscopy was used to observe ultrastructural changes in the BBB. qPCR was used to detect changes in the expression of TJs such as *Occludin* and *ZO-1* in the brain tissue of offspring rats (primer sequences are listed in Table 1). The study aimed to discuss the impact of Nd_2_O_3_ exposure on the BBB permeability of offspring rats and its mechanism. Furthermore, relevant issues regarding the damage caused by Nd_2_O_3_ to the integrity of the BBB in the offspring rat brain were explored.

**Table 1:** Primer sequences of genes.

Gene	Forward (5′-3′)	Reverse (5′-3′)
*Occludin*	GGTGCCATAGAATGAGATGTTGGA	CCAATGGGCACACCCTGATAC
*ZO-1*	TTCATCGGTGAAGTAGCCACCA	GACATTAAGGCAGCATCCAGCA
*Hrt3a*	AACAAGACTGATGACTGCTCAG	GATGGAGGATAGCTCTTGCAAG
*Hrt4*	AGGTCCGTGGAGAAGGTCGTG	CACAGCCACCATCACCAGCAG
*Sst*	CCAACCAGACAGAGAATG	ACAGGATGTGAATGTCTTC
*β-actin*	CCTAAGGCCAACCGTGAAAA	CAGAGGCATACAGGGACAACAC

#### 2.2.2 Behavioral experiments in offspring rats

Five randomly selected offspring rats from pregnant rats exposed to 0.5% Nd_2_O_3_, 1% Nd_2_O_3_, 2% Nd_2_O_3_, 2% Nd_2_O_3_ + BTVT, and the control group (distilled water of equal volume) were subjected to relevant behavioral experiments on PND43. The experiments primarily included the Morris water maze test, aiming to investigate changes in learning and memory functions in offspring rats induced by Nd_2_O_3_ exposure. The Morris water maze consists of two parts: a spatial navigation part for the first 5 days and a spatial exploration part for the 6th day, reflecting the learning and memory ability of the offspring rats. The first 5 days constituted the training period, with the maze being circular and divided into four quadrants, and a small platform placed in one of the quadrants. The offspring rats entered the pool from fixed entry points in each quadrant, and the time taken to find the platform, i.e., the escape latency, was recorded. The mean value was calculated (capped at 60 s if it exceeded 60 s). On the 6th day, the platform was removed, and the offspring rats were once again placed in the pool from the fixed entry points in the four quadrants, and the number of times the offspring rats crossed the platform area within 60 s was recorded. The mean value was calculated.

#### 2.2.3 Cortical damage in offspring rats

HE staining was used to observe changes in the quantity and shape of nerve cells in the cortical region of the rat brain, aiming to explore the pathological basis of cognitive function changes induced by Nd_2_O_3_ exposure in offspring rats.

### 2.3 Nd_2_O_3_-induced intestinal damage of offspring rats

#### 2.3.1 Changes in the intestinal flora

Fecal DNA from experimental animals was extracted, and gene sequencing was performed on the 16S rRNA of offspring rats in the Nd_2_O_3_ exposed group and the non-Nd_2_O_3_ exposed group. PCR amplification and library construction of the target segments were conducted, followed by sequencing and bioinformatics analysis using the Greengenes database. The QIIME2 software was used with the classify-sklearn algorithm to annotate species using a pre-trained Naive Bayes classifier. Alpha diversity and beta diversity assessment of the abundance of the two groups were measured using various indices such as Chao1, Observed species, Shannon, Simpson, Faith’s PD, Pielou’s evenness, and Good’s coverage. Principal coordinate analysis (PCoA) was used to analyze beta diversity, and statistical tests like PERMANOVA, anosim, and permdisp were employed for validation, exploring the impact of Nd_2_O_3_ exposure on the diversity and abundance of intestinal flora. Random forest analysis with LDA and OPLS-DA was used to identify biomarkers. Community analysis was conducted using a Venn diagram to compare the members and shared ASV/OTU numbers among different groups.

#### 2.3.2 Alterations in the intestinal mucosal barrier

Macroscopic observation included measuring intestinal length, surface color, and integrity changes, while histological observations with HE staining and AB-PAS staining focused on inflammatory cell infiltration, villi, and goblet cell alterations. AB-PAS staining colored goblet cells in the intestine, where the colors represented different substances secreted by the cells. The thickness of the mucous layer was measured at five locations on each slide and averaged. The study discussed the pathological changes in the offspring rat intestines due to Nd_2_O_3_ exposure.

#### 2.3.3 Impact on SCFAs in the intestines

Fecal samples from experimental animals were analyzed using GC–MS to detect changes in acetic acid, propionic acid, butyric acid, valeric acid, isovaleric acid, and isobutyric acid. The study explored how changes in the intestinal flora due to Nd_2_O_3_ exposure affected microbial metabolism.

### 2.4 Nd_2_O_3_-induced the BGA damage

Detection of intestinal neurogenic markers: Using *β*-actin as an internal reference, RT-PCR was employed to determine the expression of major intestinal neurotransmitters *Hrt3a* and *Hrt4*, as well as intestinal peptide *Sst* (primer sequences are listed in Table 1). This exploration aims to provide a theoretical basis for understanding the imbalance of the BGA due to Nd_2_O_3_ exposure.

### 2.5 Statistical methods

Experimental data were expressed as mean ± standard deviation and analyzed using SPSS 25.0 statistical software. When the data followed a normal distribution, comparisons of means or one-way analysis of variance (ANOVA) were conducted. When the data did not follow a normal distribution, the Mann–Whitney U test was employed. A significance level of *p* < 0.05 was set to indicate statistical significance.

## 3 Results

### 3.1 Mechanism of neurological damage induced by Nd_2_O_3_ exposure

#### 3.1.1 Detection of neodymium content in the brains of offspring rats

**Figure 1:**
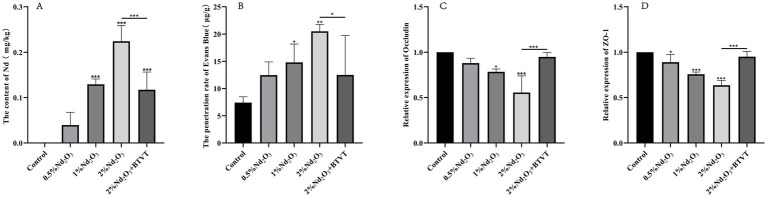
Detection of offspring rats exposed to different Nd concentrations in brain tissue and related factors. **(A)** Evans Blue permeability. **(B)** Bar graph of Nd concentration in brain tissue. **(C)** *Occludin* content. (D) *ZO-1* content. *p < 0.05, **p < 0.01, ***p < 0.001.

Neodymium content in the brains of offspring rats was measured using ICP-MS. By comparing with the control group, it was observed that as the concentration of parental Nd_2_O_3_ increased (0.5–2%), the Nd content in the brain tissues of the offspring rats significantly increased (*p* < 0.05). After intervention with Lactobacillus, the Nd content notably decreased (*p* < 0.05) (see Figure 1A). This indicates that parental rat exposure to Nd_2_O_3_ can lead to Nd accumulation in offspring rats, and Lactobacillus can reduce the accumulation of Nd in offspring.

#### 3.1.2 Observing changes in blood–brain barrier permeability in offspring rats

Using the Evans Blue method, we found that the permeability of Evans Blue in offspring PND43 (*n* = 3) gradually increased with increasing concentrations of maternal Nd_2_O_3_ (*p* < 0.05). However, following Lactobacillus intervention (2% Nd_2_O_3_ + BTVT group), there was a significant decrease in Evans Blue permeability (*p* < 0.05) (see Figure 1B). This suggests that maternal exposure to Nd_2_O_3_ can increase blood–brain barrier permeability in offspring rats, while probiotics reduce its impact on blood–brain barrier permeability.

#### 3.1.3 BBB related factors detection

Detecting the expression changes of TJs (*Occludin*, *ZO-1*) in offspring rat brain tissues. Among them, there was no significant difference in the content of *Occludin* between the control group and the 0.5% Nd_2_O_3_ group (*p* > 0.05). However, there were significant differences in the comparison between the other groups (*p* < 0.05). As the concentration of Nd_2_O_3_ increased, the content of *Occludin* decreased. After intervention with Lactobacillus, the content of Nd_2_O_3_ increased (*p* < 0.05) (see Figure 1C).

Comparing the content of *ZO-1* among groups, except for the 2% Nd_2_O_3_ + BTVT group, which showed no significant difference compared to the control group (*p* > 0.05), there were significant differences in the remaining groups (*p* < 0.05). Moreover, as the concentration of Nd_2_O_3_ increased, the content of *ZO-1* gradually decreased. After intervention with Lactobacillus, the content of *ZO-1* in offspring rats treated with 2% Nd_2_O_3_ significantly increased (*p* < 0.01) (see Figure 1D).

#### 3.1.4 BBB ultrastructural changes

**Figure 2:**
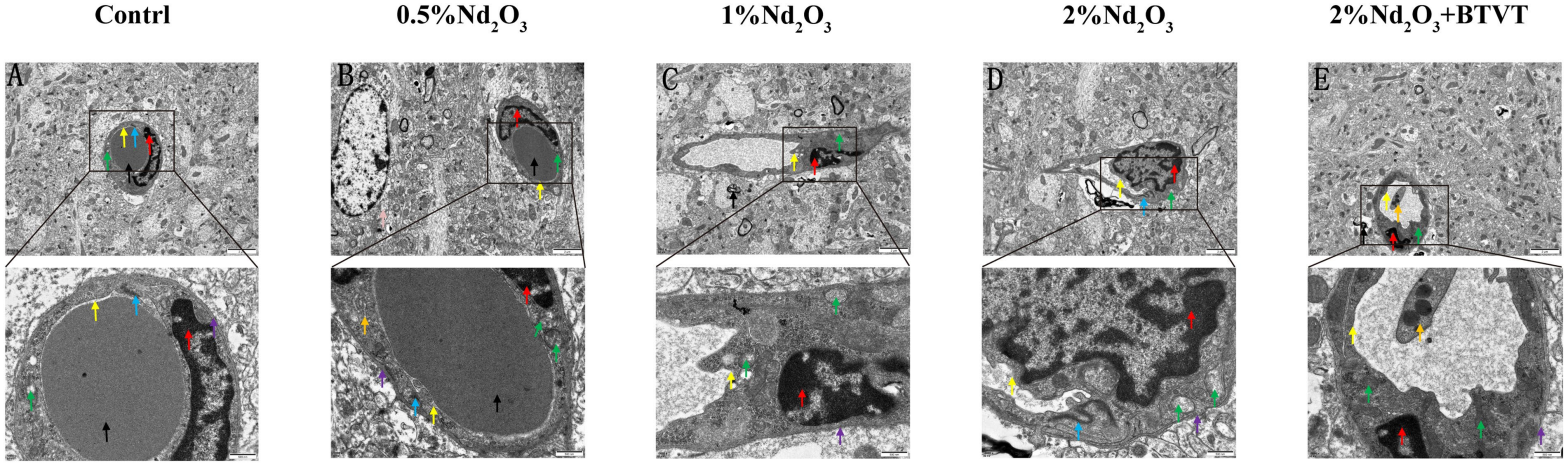
Ultrastructural observations of brain tissue under electron microscopy. (A) Control group. (B) 0.5% Nd_2_O_3_ group. (C) 1% Nd_2_O_3_ group. (D) 2% Nd_2_O_3_ group. (E) 2% Nd_2_O_3_ + BTVT group. Red arrows indicate: endothelial cell nucleus structure. Purple arrows indicate: basement membrane structure. Green arrows indicate: mitochondrial structure. Blue arrows indicate: tight junction structure. Yellow arrows indicate: cell membrane structure. Black arrows indicate: perivascular region. Pink arrows indicate: astrocyte. Orange arrows indicate: platelet.

Observation of BBB ultrastructure in 5 groups by transmission electron microscopy revealed that, compared to the negative control group, with the increase in Nd_2_O_3_ concentration (0.5–2%), the blood–brain barrier structure gradually became irregular. Some areas showed unclear basal membrane structure, local thinning, and rupture. Endothelial cells exhibited swelling, irregular nuclear morphology, significant increase in heterochromatin within the nucleus, swelling of some mitochondria in the cytoplasm, decreased electron density of the matrix with vacuolization, and fragmentation or disappearance of mitochondrial cristae. Tight junction structures were clear, cell membrane structures were blurry, lumens were narrowed, surfaces were not smooth, and partial demyelination was observed around blood vessels, indicating obvious demyelination. After addition of 2% Nd_2_O_3_ with probiotics, the blood–brain barrier structure within the field of view became regular, mitochondria in the cytoplasm appeared oval or short rod-shaped, the double-layer membrane structure was intact, and the ridge was vague. The cell membrane structure was intact, the surface was relatively smooth, the lumen was rounded, and compared to the pure 2% Nd_2_O_3_ group, the damage to the blood–brain barrier was significantly reduced (see Figure 2).

#### 3.1.5 Morris water maze experiment

**Figure 3:**
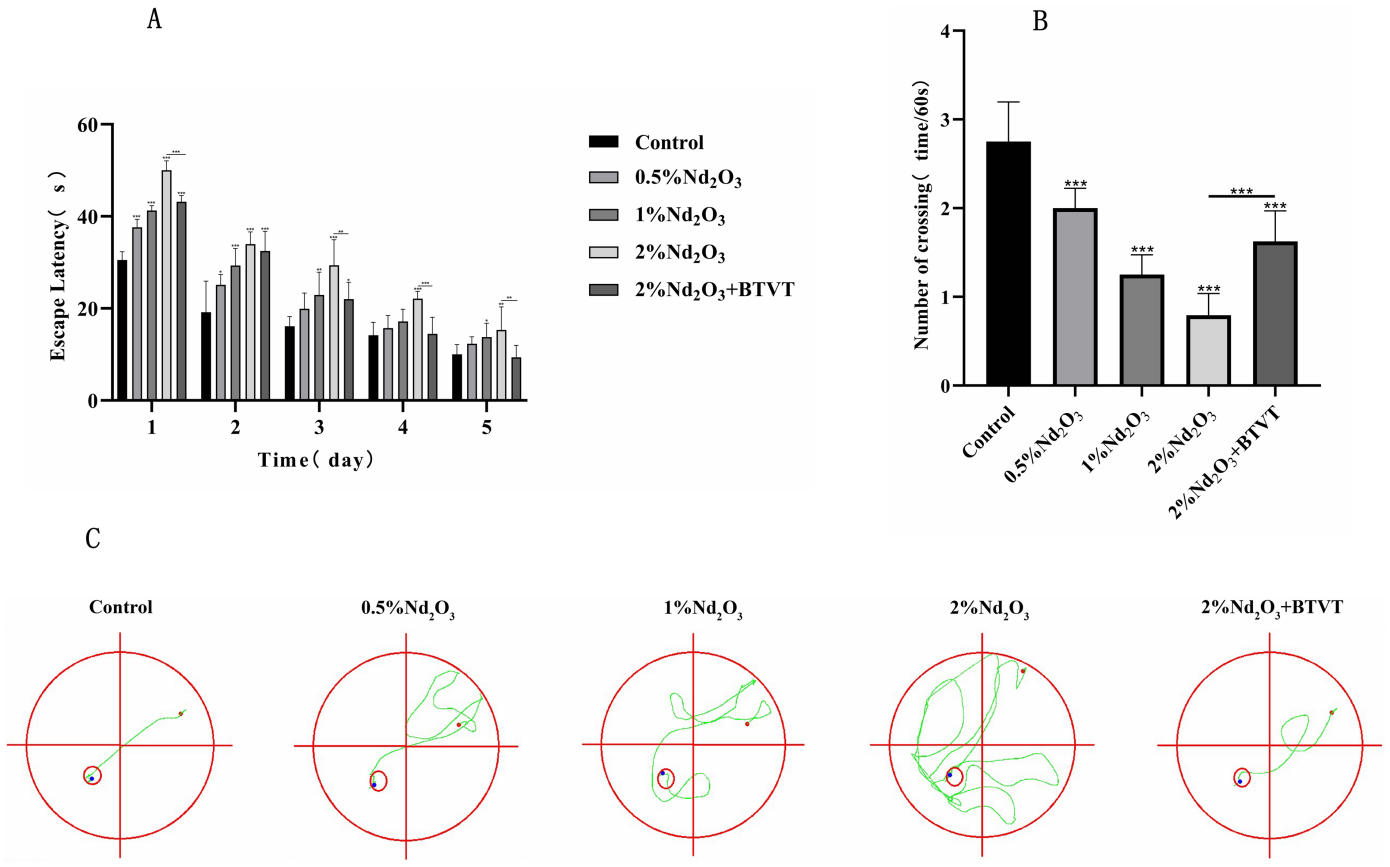
Morris water maze experiment of each group of offspring rats. **(A)** Escape latency, **(B)** number of crossings, **(C)** trajectory of crossing. *p < 0.05, **p < 0.01, ***p < 0.001.

In the spatial exploration experiment, as the days progressed, the number of times each group crossed the platform decreased. As the concentration of Nd_2_O_3_ increased, the offspring rats showed more and more the escape latency, while the number of crossings decreased after Lactobacillus intervention (*p* < 0.05) (see Figure 3A). With the increase of Nd_2_O_3_ concentration, the frequency of crossing numbers decreased, and the frequency of crossing platform increased after Lactobacillus intervention (*p* < 0.05) (see Figure 3B), indicating learning, memory and special function. With the increase in Nd_2_O_3_ concentration, the trajectories of the offspring rats’ location navigation became gradually more complex, the number of deviations increased, and the areas outside the target route gradually increased. However, after adding Lactobacillus intervention, the offspring rats only deviated from the course once, returned to the predetermined course, and successfully reached the target (see Figure 3C).

#### 3.1.6 Brain tissue structure

**Figure 4:**
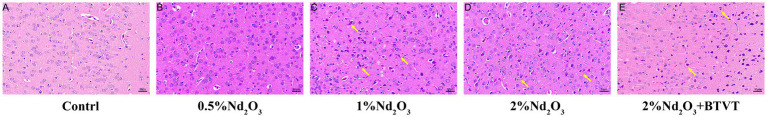
HE staining of offspring rat brain tissues in each group (200×). (A) Control group, (B) 0.5%Nd_2_O_3_ group, (C) 1%Nd_2_O_3_ group, (D) 2%Nd_2_O_3_ group, (E) 2%Nd_2_O_3_ + BTVT group. Yellow arrows indicate the denatured neuron cells.

Through observation of HE-stained brain tissue sections of the offspring rats, the control group’s brain tissue structure was basically normal. In the field of view, the neurons in the cerebral cortex were arranged regularly, abundant in number, with round nuclei, and a tight tissue structure. With the increase in Nd_2_O_3_ concentration, the tissue structure of the offspring rats gradually became abnormal. Within the field of view, the neurons in the cerebral cortex showed degeneration, deepened staining, and the cell bodies gradually became unclear (see Figure 4).

### 3.2 Mechanisms of intestinal damage induced by Nd_2_O_3_ exposure

#### 3.2.1 Sequencing of the intestinal microbiota

**Figure 5:**
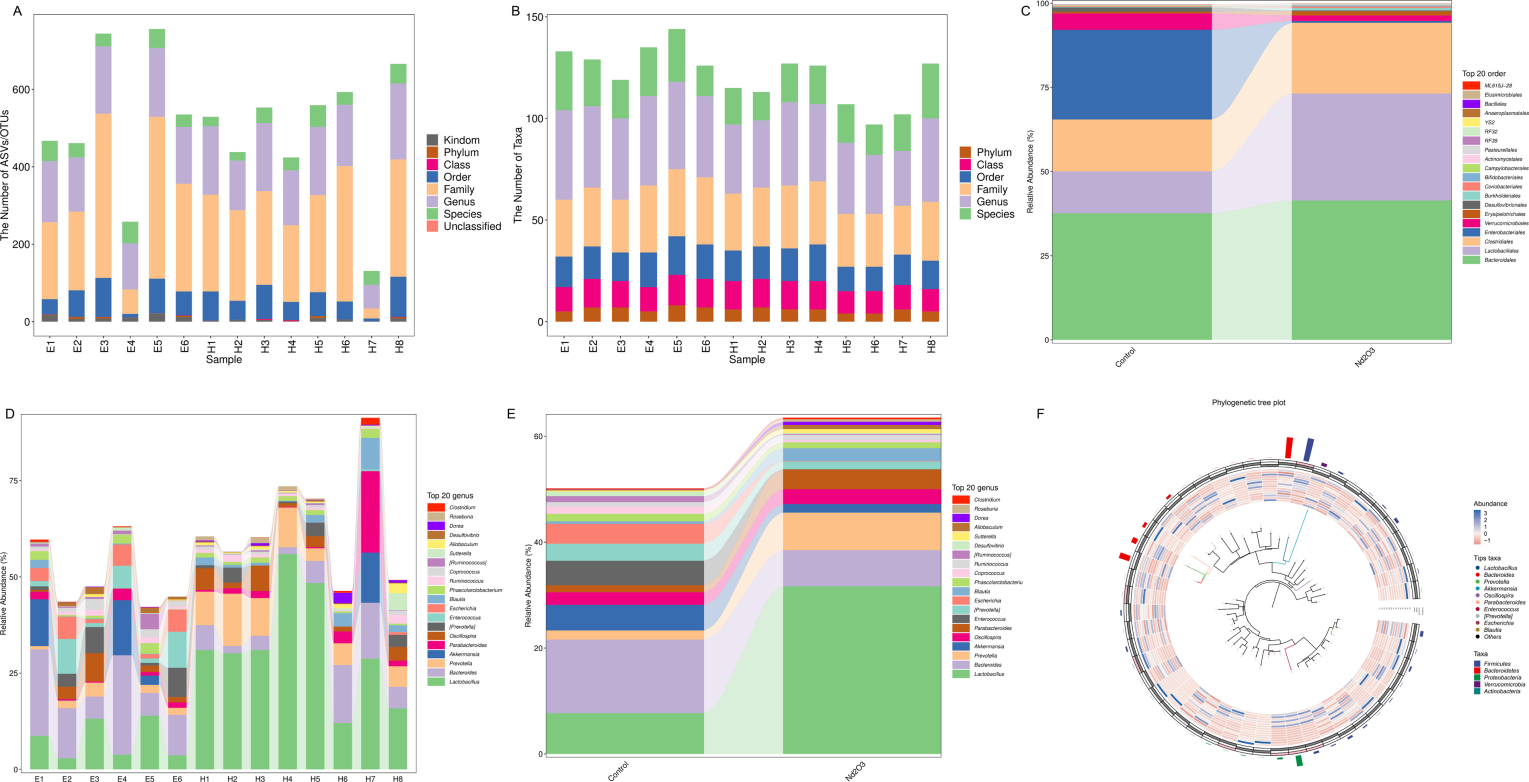
Bacterial flora resolution of offspring rats in control group and Nd_2_O_3_ group. **(A)** Bar graph of taxonomic annotation results, **(B)** Bar graph of the number of taxonomic units at each hierarchical level, **(C)** Bar graph of species composition at the order level for two groups, **(D)** Bar graph of species composition at the gene level for each sample, **(E)** Bar graph of species composition at the gene level for two groups, **(F)** Phylogenetic tree generated by GraPhlAn.

Fecal DNA was separately extracted from offspring rats in the non-Nd_2_O_3_ exposure group (Control group, E1–E6) and Nd_2_O_3_ exposure group (Nd_2_O_3_ group, H1–H8). From the perspective of ASV/OTU numbers and Taxa numbers at various classification levels in each sample, columns representing genus and species levels were relatively long, while those representing phylum level were shorter, indicating a higher resolution of annotation included (see Figures 5A,B). After parental rats were exposed to Nd_2_O_3_, the content of orders Clostridiales, Lactobacillales, and Bacteroidales in the intestinal microbiota of offspring rats increased, while the content of orders Verrucomicrobiales and Enterobacteriales decreased (see Figure 5C). At the species level, Bacteroides, Escherichia, and Akkermansia decreased, while Lactobacillus, Prevotella, Parabacteroides, and Blautia increased (see Figures 5D–F).

**Figure 6:**
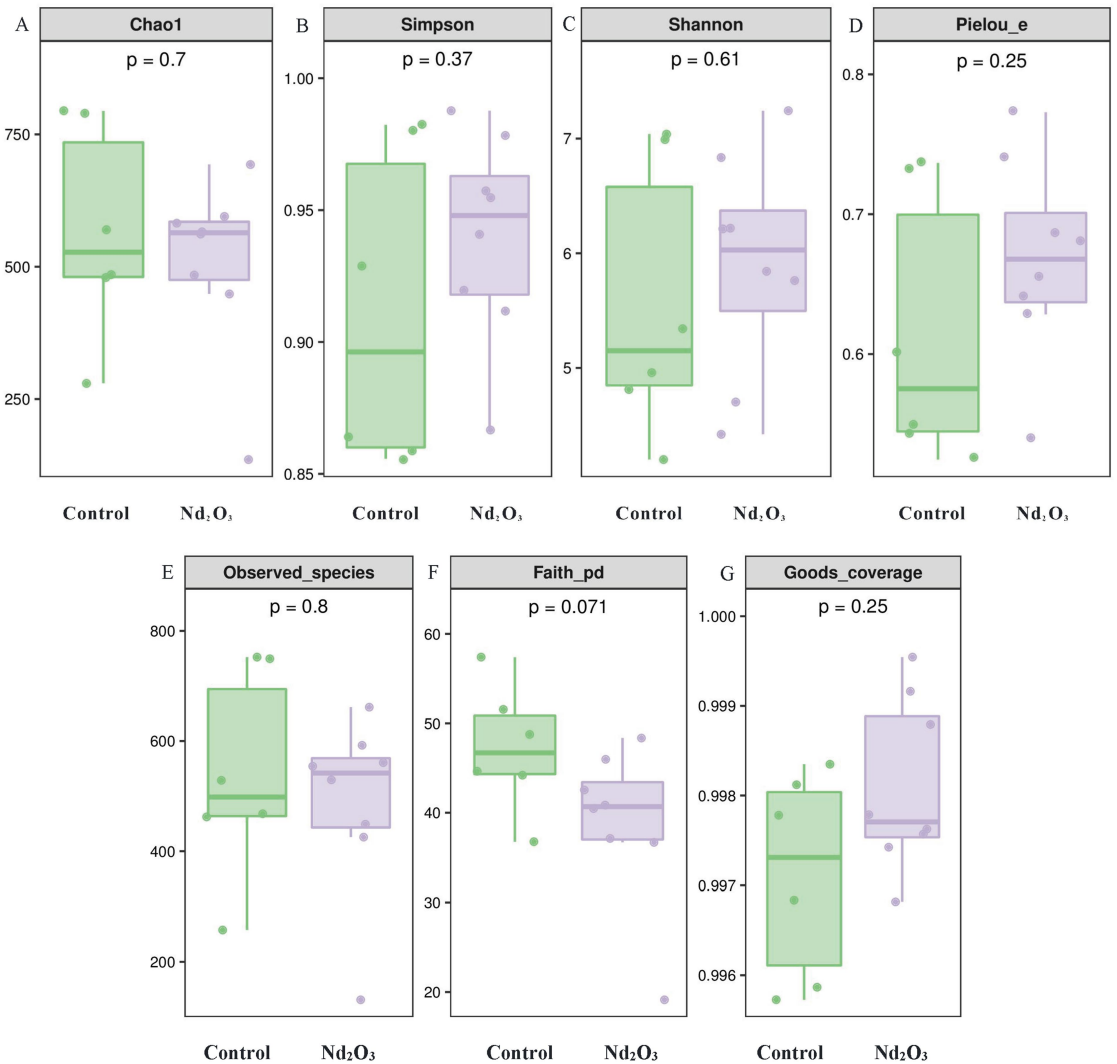
Alpha diversity of intestinal flora in offspring rats. **(A)** Comparison of Chao1 index between the control group and the Nd_2_O_3_ group, **(B)** Comparison of Simpson index between the control group and the Nd_2_O_3_ group, **(C)** Comparison of Shannon index between the control group and the Nd_2_O_3_ group, **(D)** Comparison of Pielou’s evenness index between the control group and the Nd_2_O_3_ group, **(E)** Comparison of Observed species index between the control group and the Nd_2_O_3_ group, **(F)** Comparison of Faith’s PD index between the control group and the Nd_2_O_3_ group, **(G)** Comparison of Good’s coverage index between the control group and the Nd_2_O_3_ group.

In terms of diversity, our study utilized multiple indices to evaluate the *alpha* diversity of offspring rats after Nd_2_O_3_ exposure from various dimensions. In this research, only Faith’s PD index showed statistical significance between the two groups (*p* < 0.05) (see Figure 6F), indicating differences in evolution-based alpha diversity between the two groups. However, the indices for Chao1, Simpson, Shannon, Pielou’s evenness, Observed species, and Good’s coverage showed no statistical significance (*p* > 0.05) (see Figures 6A–E,G), suggesting no significant differences in richness, evenness, and coverage between the two groups.

**Figure 7:**
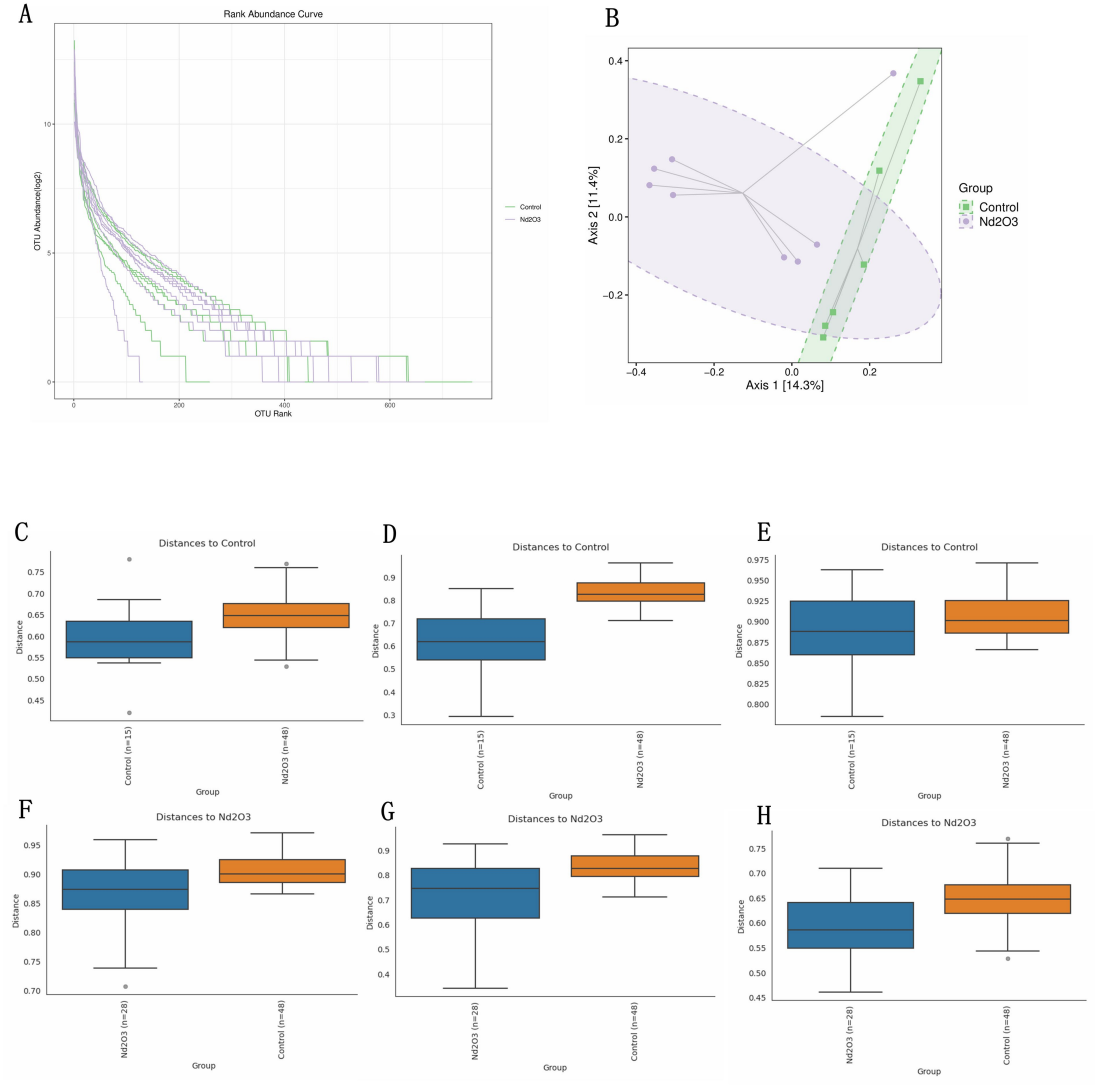
Beta diversity of intestinal flora in offspring rats. (A) OTU rank abundance curve of two groups. (B) The distribution of two groups of samples on the sequential ordering axis. (C–H) Beta diversity results validation. (C,F) Pairwise anosim results, (D,G) pairwise permanova results, (E,H) pairwise permdisp results.

PCoA was employed to reduce the dimensionality of multidimensional microbiota data (see Figure 7A). Analysis of the distribution along the principal axis revealed significant differences in beta diversity between the two groups of samples (Control and Nd_2_O_3_ groups) (see Figure 7B). Through PCoA analysis of the multidimensional microbial data and subsequent validation using PERMANOVA, anosim, and permdisp., it was found that there were differences in beta diversity between the two groups (all three validation results showed *p* < 0.05) (see Figures 7C–H).

**Figure 8:**
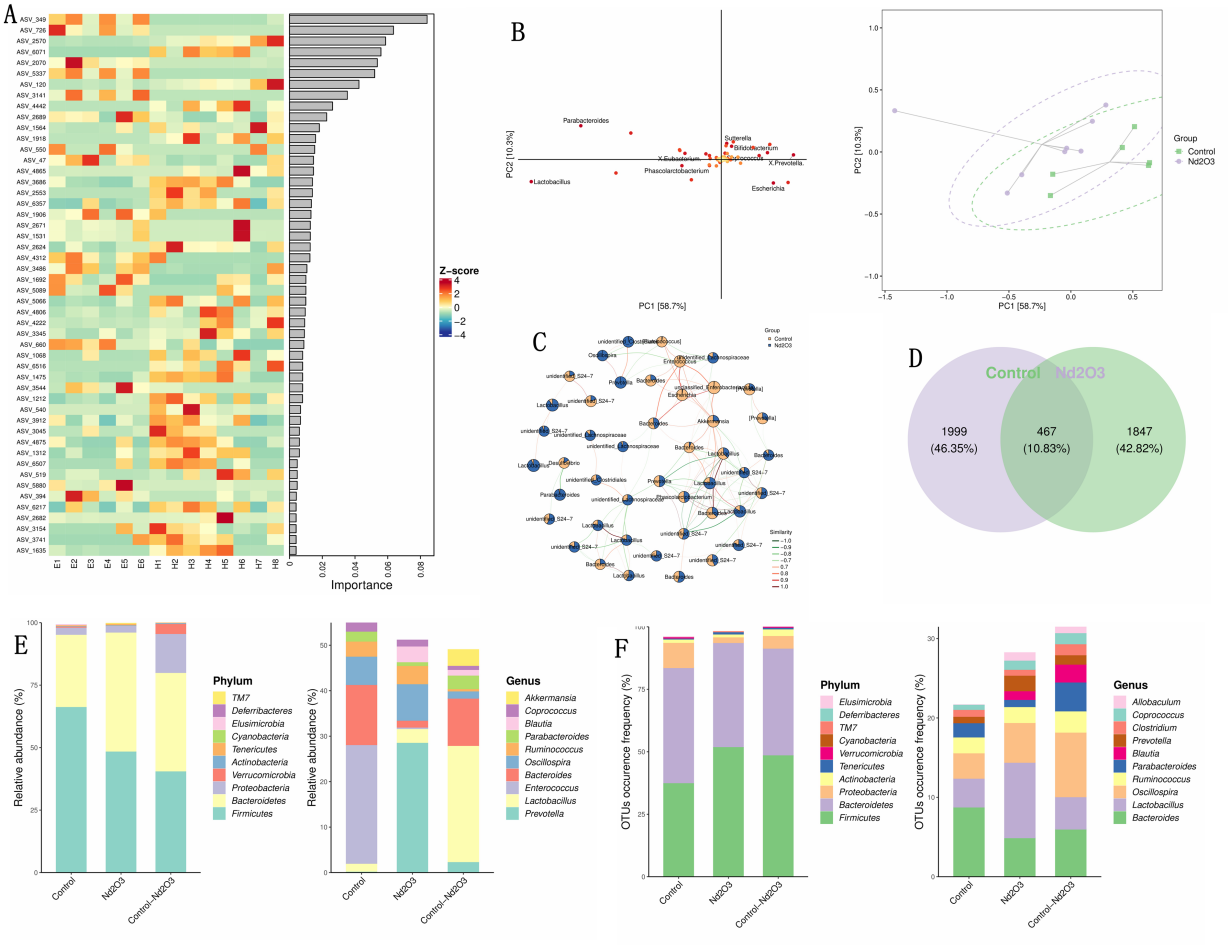
Correlation between control and Nd_2_O_3_ groups. (A) Heatmap from random forest analysis of the top 100 important ASVs/OTUs/taxa. (B) OPLS-DA ellipse of control and Nd_2_O_3_ groups. (C) Subnetwork of control and Nd_2_O_3_ groups. (D) Venn plot of control and Nd_2_O_3_ groups. (E) Bar graph showing the number of ASVs/OTUs in different regions of the Venn diagram. (F) Bar graph showing the abundance of ASVs/OTUs in different regions of the Venn diagram.

Using LDA and OPLS-DA for random forest analysis screening, ASV_349 was identified as the most important inter-group differentiating bacterial species (see Figures 8A,B). The sub network of Control and Nd_2_O_3_ groups shows the gene with the highest similarity between the two groups is Lactobacillus (see Figure 8C). The similarity of the two groups Community analysis using a Venn diagram revealed that the proportion of shared bacterial species between the two groups was 10.83% (467 species) (see Figure 8D). In terms of both the number and abundance values of ASV/OTU, Bacteroidetes and Firmicutes were the highest (see Figure 8E). In quantitative terms, the content of Lactobacillus was the highest in both groups, and the top five abundant species were Oscillosa, Bacteroides, Lactobacillus, Ruminococcus, and Parabacteroides (see Figure 8F).

#### 3.2.2 Alterations of the intestinal mucosal barrier

**Figure 9:**
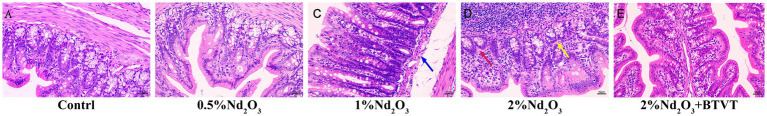
PND 43d colon HE staining. (A) control group, (B): 0.5%Nd_2_O_3_ group, (C) 1%Nd_2_O_3_ group, (D) 2%Nd_2_O_3_ group, (E) 2%Nd_2_O_3_+ BTVT group. Blue arrow indicates: Increased submucosal space and mild edema. Yellow arrow indicates: Reduced number of crypts with inflammatory cell infiltration. Red arrow indicates: Cell necrosis, nucleus fragmentation and hyperpyretic staining.

**Figure 10:**
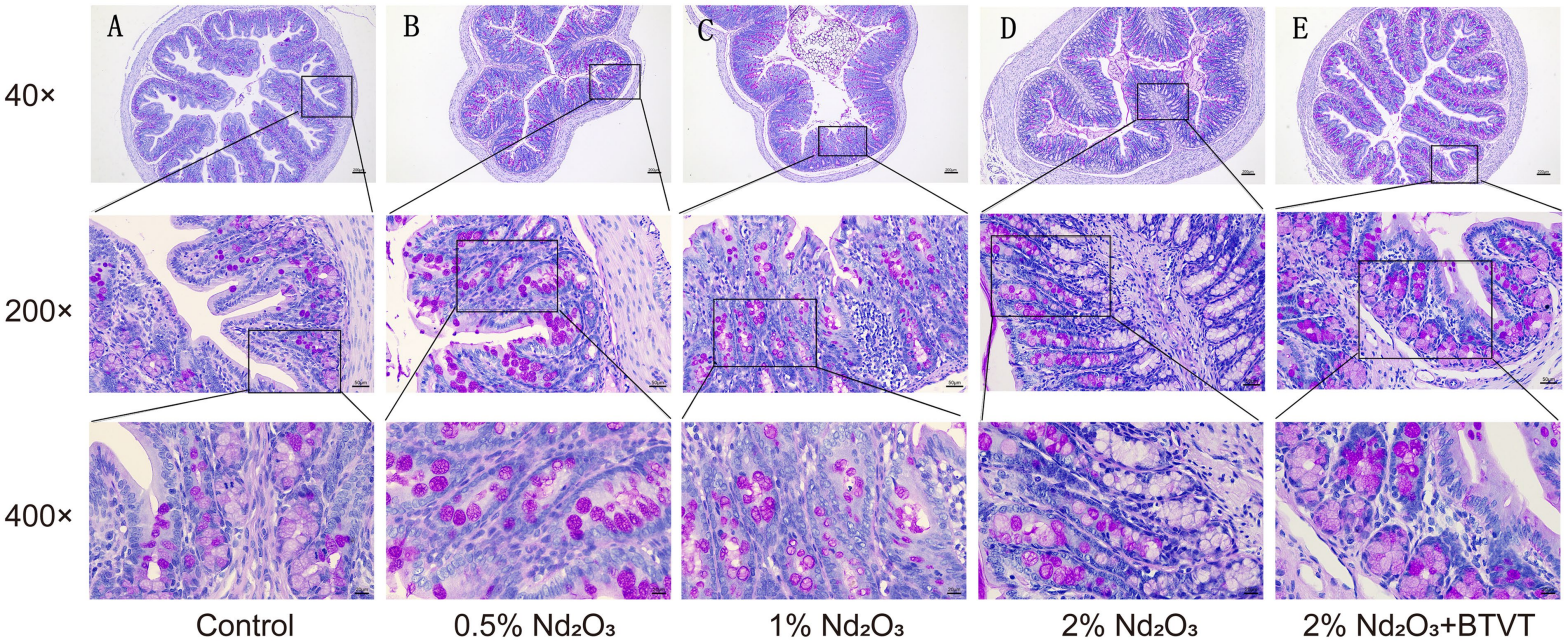
PND 43d Rat Colon AB-PAS Staining: As Nd_2_O_3_ concentration increases, the number of goblet cells (stained pink with AB-PAS) gradually decreases among the groups. Following intervention with probiotics, there is no significant change in the number of goblet cells in the colon of rats treated with 2% Nd_2_O_3_. Post-intervention with Nd_2_O_3_, offspring rat intestinal mucus thickens, which is attenuated with probiotic intervention, resulting in thinner mucus. (A) Control group, (B) 0.5% Nd_2_O_3_ group, (C) 1% Nd_2_O_3_ group, (D) 2% Nd_2_O_3_ group, (E) 2% Nd_2_O_3_ + BTVT group.

After dissecting the offspring rats on PND 43d, observations were made regarding the length and surface color changes of the colon. Regardless of Nd_2_O_3_ intervention, there were no significant differences observed in the length or surface color of the colon. Upon selecting and fixing colonic segments for each group and staining with HE, it was found that with increasing Nd_2_O_3_ concentration, the colonic tissue structure gradually became abnormal, lymph nodes enlarged, crypt numbers decreased, and were accompanied by a small amount of inflammatory cell infiltration. However, the epithelial cells of the mucosal layer were closely arranged and not shed. Following intervention with Lactobacillus after Nd_2_O_3_ exposure, the colonic tissue structure of the offspring rats was essentially normal. The epithelial cells of the mucosal layer were closely arranged without shedding in the field of view, the crypt structure was intact and orderly, and there was no apparent infiltration of inflammatory cells in the tissue (see Figure 9). After AB-PAS staining, it was discovered that with increasing Nd_2_O_3_ concentration, the number of goblet cells in each group gradually decreased, showing a significant difference (*p* < 0.05). The number of goblet cells in the 2% Nd_2_O_3_ + BTVT group did not show a significant difference compared to the pure 2% Nd_2_O_3_ group (*p* > 0.05). In comparison to the control group, the thickness of the colonic mucus layer increased significantly in all groups exposed to Nd_2_O_3_ (*p* < 0.05), but there was no significant difference between the different Nd_2_O_3_-exposed groups (*p* > 0.05). The addition of Lactobacillus intervention resulted in a thinner colonic mucus layer compared to the Nd_2_O_3_-exposed group (*p* < 0.05), with no significant difference compared to the control group (*p* > 0.05) (see Figure 10).

#### 3.2.3 Affection of the SCFAs in the intestines

**Figure 11:**
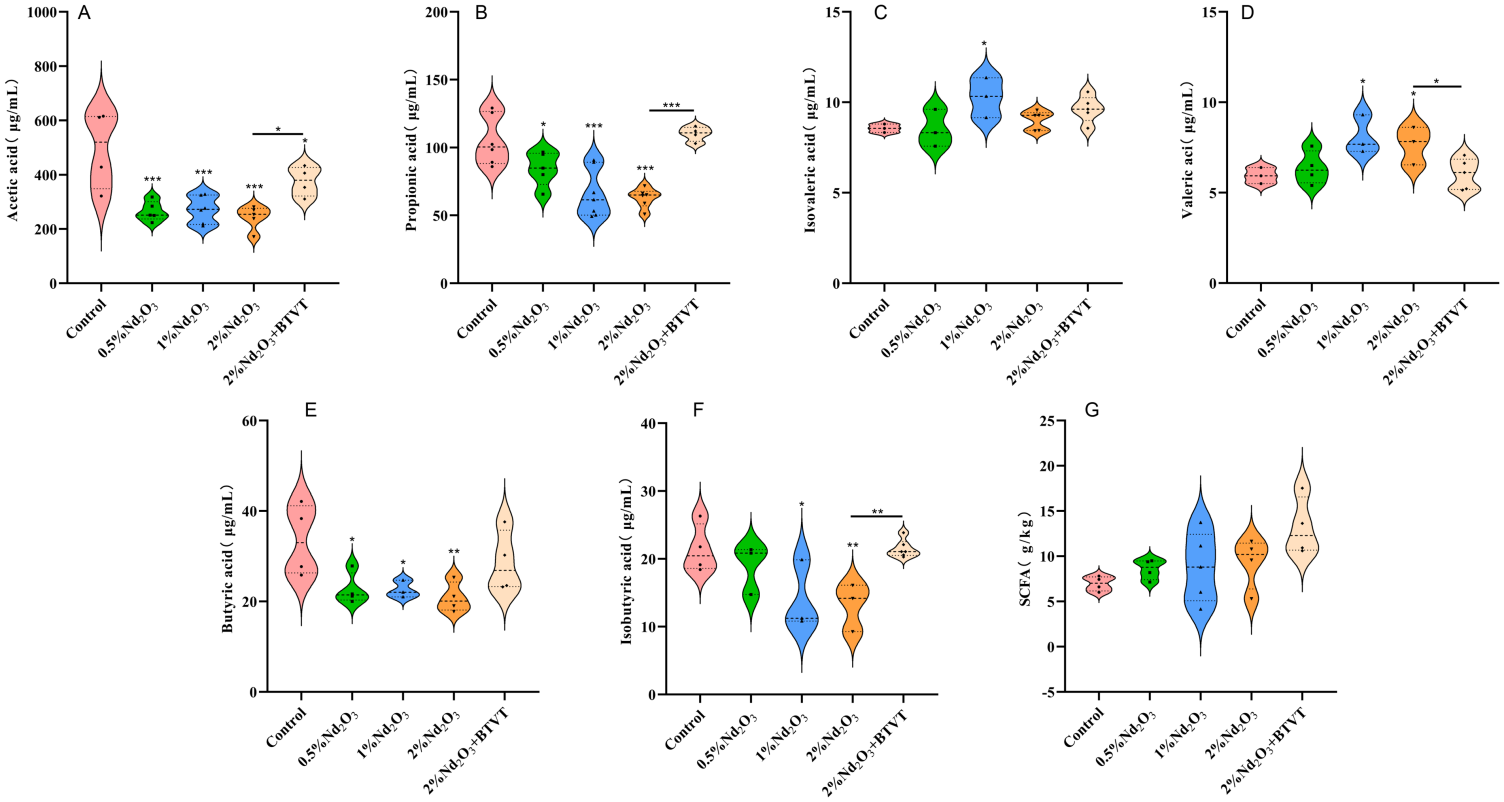
Changes in SCFAs (short-chain fatty acids) in the intestines of offspring rats exposed to various concentrations of Nd_2_O_3_. **(A)** Acetic acid content, **(B)** propionic acid content, **(C)** butyric acid content, **(D)** valeric acid content, **(E)** isovaleric acid content, **(F)** isobutyric acid content, **(G)** total SCFAs content. *p < 0.05, **p < 0.01, ***p < 0.001.

The feces of experimental animals were extracted, and GC–MS was used to detect changes in the content of acetic acid, propionic acid, butyric acid, valeric acid, isovaleric acid, and isobutyric acid in the feces. It was found that after Nd_2_O_3_ intervention, the acetic acid content decreased significantly (*p* < 0.01). There was no significant difference in acetic acid content with increasing Nd_2_O_3_ concentration (*p* > 0.05), and the addition of Lactobacillus increased the acetic acid content in the offspring rats exposed to Nd_2_O_3_ (*p* < 0.05) (see Figure 11A). After Nd_2_O_3_ intervention, the propionic acid content decreased (*p* < 0.01), and with increasing Nd_2_O_3_ concentration, the propionic acid content significantly decreased (*p* < 0.05). The addition of Lactobacillus increased the propionic acid content in the offspring rats exposed to Nd_2_O_3_ (*p* < 0.05) with no significant difference compared to the control group (*p* > 0.05) (see Figure 11B). After Nd_2_O_3_ intervention, the butyric acid content decreased (*p* < 0.05), and there was no significant difference in butyric acid content with increasing Nd_2_O_3_ concentration (*p* > 0.05). The addition of Lactobacillus increased the butyric acid content in the offspring rats exposed to 2% Nd_2_O_3_ (*p* < 0.05) with no significant difference compared to the control group (*p* > 0.05) (see Figure 11C). Compared to the control group, there was no significant difference in valeric acid content after intervention with 0.5% Nd_2_O_3_ (*p* > 0.05), while the valeric acid content increased after intervention with 1% Nd_2_O_3_ and 2% Nd_2_O_3_ (*p* < 0.05). There was no significant difference between the two, and the addition of Lactobacillus resulted in a decrease in valeric acid content in the offspring rats exposed to 2% Nd_2_O_3_, with no significant difference compared to the control group (see Figure 11D). There was no significant change in isovaleric acid content in the intestines of offspring rats after intervention with 0.5% Nd_2_O_3_ (*p* < 0.05). After intervention with 1% Nd_2_O_3_, the isovaleric acid content increased (*p* > 0.05), however, there was no significant change in isovaleric acid content compared to the control group after intervention with 2% Nd_2_O_3_ (*p* > 0.05). Additionally, the addition of Lactobacillus did not result in a significant difference in isovaleric acid content in the intestines of the offspring rats exposed to 2% Nd_2_O_3_ compared to the group exposed to 2% Nd_2_O_3_ without Lactobacillus intervention (*p* > 0.05) (see Figure 11E). There was no significant difference in isobutyric acid content in the intestines of the offspring rats after intervention with 0.5% Nd_2_O_3_ compared to the control group (*p* > 0.05). The isobutyric acid content decreased after intervention with 1% Nd_2_O_3_ and 2% Nd_2_O_3_ (*p* < 0.05), and the addition of Lactobacillus increased the isobutyric acid content in the offspring rats exposed to 2% Nd_2_O_3_ (*p* < 0.05), with no significant difference compared to the control group (*p* > 0.05) (see Figure 11F). There was no significant difference of total SCFAs content among the five groups (*p* > 0.05) (see Figure 11G).

### 3.3 The impact of Nd_2_O_3_ exposure on the BGA

**Figure 12:**
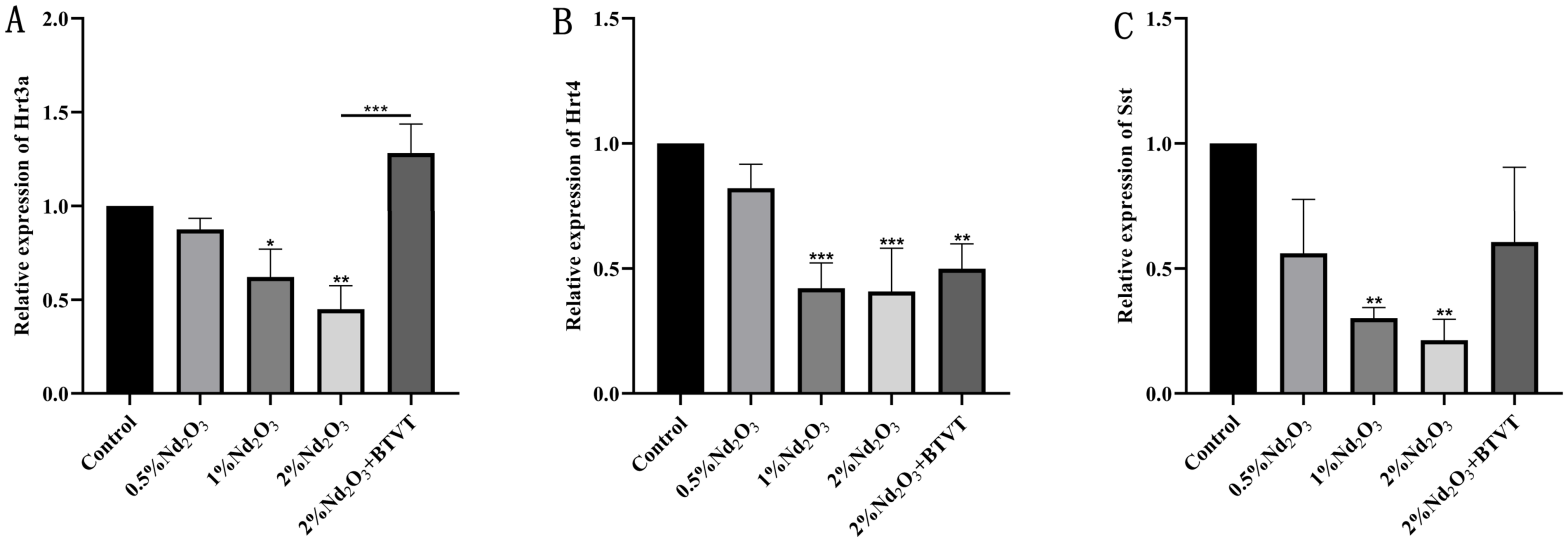
mRNA expression of intestinal neurotransmitters and neuropeptides in offspring rats following exposure to different concentrations of Nd_2_O_3_. **(A)** Expression of *Hrt3a* in offspring rats after exposure to different concentrations of Nd_2_O_3_. **(B)** Expression of *Hrt4* in offspring rats after exposure to different concentrations of Nd_2_O_3_. **(C)** Expression of *Sst* in offspring rats after exposure to different concentrations of Nd_2_O_3_. *p < 0.05, **p < 0.01, ***p < 0.001.

Comparison of mRNA expression levels of *Hrt3a*, *Hrt4*, and *Sst* among different groups: The expression level of *Hrt3a*, except for no significant difference between the control group and the 0.5% Nd_2_O_3_ group (*p* > 0.05), showed significant differences in pairwise comparisons between the other groups (*p* < 0.05) (see Figure 12A). For the expression level of *Hrt4*, there was no significant difference between the control group and the 0.5% Nd_2_O_3_ group (*p* > 0.05). In pairwise comparisons, there were no significant differences between the 1% Nd_2_O_3_ group, 2% Nd_2_O_3_ group, and 2% Nd_2_O_3_ + BTVT group (*p* > 0.05), while significant differences were observed in pairwise comparisons between the remaining groups (*p* < 0.05) (see Figure 12B). As for the expression level of *Sst*, except for no significant difference between the 0.5% Nd_2_O_3_ group and the 2% Nd_2_O_3_ + BTVT group (*p* > 0.05), significant differences were observed in pairwise comparisons between the other groups (*p* < 0.05) (see Figure 12C). This indicates that the mechanism of BGA damage caused by Nd_2_O_3_ exposure is inversely correlated with the mRNA expression of intestinal neurotransmitters *Hrt3a*, *Hrt4*, and *Sst*, and the exposure of parental rats to Nd_2_O_3_ can promote the expression of *Hrt3a* and *Sst* in offspring rats after intervention with Lactobacillus.

## 4 Discussion

### 4.1 Nd_2_O_3_’s destruction of the BBB

Research has shown that increased BBB permeability is a critical threshold for toxic and harmful factors in the environment to enter the central nervous system and cause neurodevelopmental damage. For example, when pregnant and lactating rats drink water containing REEs, the brains of their offspring can exhibit rare earth accumulation that is several times higher than that of the control group (16). When male Sprague–Dawley rats were intravenously injected with 10 mg/kg body weight of neodymium chloride (NdCl_3_), dissection after 7 days revealed significant absorption and retention of neodymium in the rat brain, indicating that neodymium can cross the BBB, and the brain has an accumulative effect on neodymium (17). The above studies indicate that Nd can penetrate the BBB, thereby causing damage to the nervous system. Therefore, the brain serves as both a storage organ and a target organ for neodymium damage. Furthermore, our research has found that exposure of parental rats to Nd_2_O_3_ can reduce the content of TJs in the offspring rats, increase the BBB permeability in the offspring rats, leading to neodymium accumulation in the offspring rats, and this accumulation is positively correlated with the concentration of Nd_2_O_3_. However, after the intervention of probiotics, the content of TJs in the BBB of the offspring rats increased, the BBB permeability decreased, and likewise, the accumulation of neodymium in the offspring rats decreased. After staining the brain tissue of the offspring rats with HE, it was found that the inflammation of the brain tissue gradually intensified after parental Nd_2_O_3_ exposure, but the addition of probiotics reduced the inflammation in the brain tissue of the offspring rats. The above results indicate that exposure of parental rats to Nd_2_O_3_ can lead to reduced content of TJs in the offspring, increased BBB permeability, neodymium accumulation, occurrence of brain tissue inflammation, and thus affect the neurodevelopment of the offspring. However, probiotics can alleviate the aforementioned effects. The Morris water maze experiment revealed that exposure of parental rats to Nd_2_O_3_ can lead to reduced spatial orientation ability in the offspring rats. However, after parental rats were intervened with Nd_2_O_3_ with BTVT, there was no significant change in the spatial orientation ability of the offspring rats compared to the control group, indicating that the intervention of probiotics in parental rats can mitigate the effect of Nd_2_O_3_ on the spatial orientation ability of the offspring rats.

### 4.2 Destruction of the intestinal barrier by Nd_2_O_3_

The intestinal epithelial barrier consists of tightly connected basal layer cells and a dynamic mucus layer containing secretory IgA (sIgA) and antimicrobial peptides. The main function of the intestinal epithelial barrier is to regulate the absorption of nutrients, electrolytes, and water from the lumen into circulation, prevent pathogenic microorganisms and luminal toxins from entering, regulate molecular exchange between the environment and the host through the intestinal barrier, and affect the balance between tolerance to self and non-self antigens and immunity (18). The permeability of the intestinal barrier can be influenced by inflammatory mediators and activity of the sympathetic nervous system (19). Metabolites produced by gut microbiota in the intestine can interact with immune cells to influence host health, and these metabolites can significantly affect the integrity of the intestinal epithelial barrier and regulate the maintenance of intestinal homeostasis (20). Existing research shows that heavy metals promote oxidative stress, thereby altering the permeability of the intestinal barrier, leading to inflammation, and facilitating the absorption of heavy metals into the brain. Humans ingest various nutrients, vitamins, and active metals through contaminated food or water every day, and interventions to reduce toxic metals in the environment may reduce the inflammatory burden of the gut microbiota and the occurrence of potential neurological disorders (21). The global industrial influx of heavy metals into the environment is associated with brain pathology from environmental sources, suggesting that initial environmental exposures may interact with gut-related microbiota before reaching the brain. However, research on the damage to the intestinal epithelial barrier caused by low-dose, long-term exposure to rare earth elements is limited. In this study, as the concentration of parental Nd_2_O_3_ exposure increased, mucosal inflammation in the offspring’s intestines gradually worsened, and the number of goblet cells decreased. After parental rats were intervened with probiotics, no significant inflammation was observed in the offspring’s intestinal mucosa, but there was no significant change in the reduction of goblet cell numbers. Parental Nd_2_O_3_ exposure resulted in thickening of the mucous layer in the offspring’s intestines, which was reversed to thinner mucus with probiotic intervention. These results suggest that the intestine may also be one of the target organs damaged by parental Nd_2_O_3_ exposure, causing injury to the intestinal mucosa and affecting childhood intestinal development.

### 4.3 Relationship between Nd_2_O_3_ and the BGA

The human intestines harbor approximately 10^13^ to 10^14^ microbes, with the gut microbiome possessing over 150 times the total number of genes found in the human genome (9). It is widely acknowledged that there is bidirectional communication and interaction between the gut and the brain (22). The enteric nervous system and the central nervous system serve as crucial bidirectional regulatory channels. The intestinal wall contains numerous sensory neurons that can perceive changes in the intestinal environment, autonomously regulating metabolic and endocrine activities within the intestines in response to physicochemical alterations, a system referred to as the enteric nervous system or the “gut-brain axis,” also known as the “second brain” of the body (23).

The gut microbiome can influence the permeability of the blood–brain barrier (BBB) by regulating the expression of tight junction proteins (24). Short-chain fatty acids (SCFAs) act as the primary signaling molecules for host-microbe communication mediated by enteroendocrine cells and intestinal enterochromaffin cells, participating in various host processes such as gastrointestinal function, blood pressure regulation, circadian rhythms, neuroimmune function, enhancing intestinal epithelial integrity, increasing mucus production, regulating intestinal motility, and exerting anti-inflammatory effects (25). There is evidence suggesting that SCFAs may serve as crucial signaling metabolites, influencing the development and maintenance of the BBB through epigenetic modifications (13).

Research has reported that SCFAs, mainly acetate, propionate, and butyrate, play a protective role in maintaining BBB integrity within the BGA (26). This protective mechanism is mediated through G-protein-coupled receptor signaling, involving the inhibition of histone deacetylase activity, interference with the signaling pathways of transcription factors NF-κB and Nrf2. Bacterial metabolites, particularly SCFAs were shown to induce tryptophan hydroxylase 1 in enterochromaffin cells to release serotonin in the gut. Serotonin can stimulate the sympathetic nervous system to influence memory and learning process. Emerging data supports the fact that dysbiosis in gut microbiota during functional GI disorders disrupts the GBA and leads to mood disorders. Experimental evidences also suggested that probiotic can induce brain derived neurotrophic factors in the hippocampus and cerebral cortex and regulate cognitive functions as well as muscle repair, regeneration, and differentiation (27). Our study found that after parental rats were exposed to Nd_2_O_3_, the feces of their offspring exhibited decreased levels of acetate, propionate, and butyrate, while there was an increase in valerate and isovalerate. However, after treatment with probiotics, the levels of acetate and propionate in the offspring increased, demonstrating that Nd_2_O_3_ can disrupt BBB integrity and affect the BGA by modulating SCFAs. The mechanism of action for Nd_2_O_3_-induced BGA damage is inversely correlated with the mRNA expression of gut-derived neurotransmitters *Hrt3a*, *Hrt4*, and BG peptide *Sst*. Additionally, after intervention with probiotics, the parental rats exposed to Nd_2_O_3_ promoted the expression of *Hrt3a* and *Sst* in their offspring.

### 4.4 The prevention and repair of Nd_2_O_3_ damage by probiotics

Early life, including the prenatal period, is a particularly important developmental window for the central nervous system, with widespread synaptogenesis and myelination occurring. It is also an important period for the development of the intestinal microbiota, as the energy produced by microorganisms plays a crucial role in brain development. During this period, exposure to different microorganisms, diets, stressors, antibiotics, and other factors not only affects communication within the developing central nervous system but also influences the structure and function of the microbiota. Through these mechanisms, various influences during early life events play a key role in programming the gut microbiota and the brain, and may be implicated in the etiology of several neurodevelopmental disorders (28).

The diversity of the gut symbiotic microbiota plays an important regulatory role in infant neurodevelopment. Research has shown that bacterial metabolic products (bacterial peptidoglycans) in the mother’s body can affect fetal neurodevelopment and cognitive function through the placenta (29), and may also disrupt the integrity of the blood–brain barrier (BBB) (23). This could lead to the infiltration of inflammatory factors and the pro-inflammatory effects of microbial-derived metabolites (such as phenylalanine or isoleucine), affecting the occurrence of diseases in offspring such as psychological, behavioral, cognitive, emotional, and neurological disorders (30).

For example, Ganal-Vonarburg et al. (31), researchers have confirmed that the mother’s gut microbiota plays a crucial role in promoting infant development and maintaining their normal physiological activities. The mother’s diet and stress exposure during pregnancy can affect the composition and function of the infant’s gut microbiota. The infant’s gut microbiota is relatively unstable in the early stages, gradually gaining stability and diversity during the growth process. Most of the infant’s gut microbiota is colonized at birth, with the mode of delivery, breastfeeding, premature birth, environment, host genetics, antibiotic exposure, infection status, and stress exposure all influencing the complex microbiota that the infant is exposed to the environment (32).

A study of 89 infants found that the composition of the gut microbiota at 1 year of age was related to cognitive performance 1 year later, further emphasizing the importance of this early period in the interaction between the gut microbiota and brain development (33).

In our study, we found that the levels of *Escherichia coli*, Akkermansia, and Bacteroides decreased, while Lactobacillus, Prevotella, Parabacteroides, and Blautia increased in the feces of offspring rats through 16S RNA gene detection. Among them, the increase in Lactobacillus was the most significant. Using random forest analysis with LDA and OPLS-DA, ASV_349 was identified as the most important inter-group differentiating bacteria. Community analysis using a Venn diagram showed that the proportion of intersecting bacteria between the two groups was 10.83% (467 species). The content of Lactobacillus was the highest among the ASV/OTU in both groups.

After intervening with the parent rats exposed to 2% Nd_2_O_3_ by adding Lactobacillus, we found significant recovery of inflammation in both brain and colon tissues, and a decrease in BBB permeability. This indicates that Lactobacillus has a dual repair effect on the damage to brain and colon tissues caused by Nd_2_O_3_ in the BGA. The specific mechanism of this action will provide direction for further research.

## 5 Conclusion

Our research showed that parental exposure to Nd_2_O_3_ during pregnancy can affect the offspring’s BGA, targeting the brain and colon tissues, leading to increased BBB permeability in offspring and affecting their neurodevelopment. It also causes damage to the intestinal mucosa, impacting children’s intestinal development. However, Lactobacillus can alleviate the aforementioned effects. These findings will provide valuable insights for understanding the neurodevelopmental and intestinal developmental toxicity of Nd_2_O_3_ and related damage, and call for a comprehensive assessment of the health risks for susceptible populations (such as pregnant women) exposed to Nd_2_O_3_.

## Data Availability

The original contributions presented in the study are included in the article, further inquiries can be directed to the corresponding authors.
